# Human Oral Epithelial Cells Suppress T Cell Function *via* Prostaglandin E2 Secretion

**DOI:** 10.3389/fimmu.2021.740613

**Published:** 2022-01-19

**Authors:** Jose L. Sanchez-Trincado, Hector F. Pelaez-Prestel, Esther M. Lafuente, Pedro A. Reche

**Affiliations:** Laboratory of Immunomedicine, Department of Immunology & O2, School of Medicine, Complutense University of Madrid, Madrid, Spain

**Keywords:** oral epithelial cells, dendritic cells, T cells, immunomodulation, PGE2, viral infection

## Abstract

The oral mucosa is constantly exposed to a plethora of stimuli including food antigens, commensal microbiota and pathogens, requiring distinct immune responses. We previously reported that human oral epithelial cells (OECs) suppress immune responses to bacteria, using H413 and TR146 OEC lines and primary OECs in co-culture with dendritic cells (DCs) and T cells (OEC-conditioned cells). OECs reduced DCs expression of CD80/CD86 and IL-12/TNFα release and impaired T cell activation. Here, we further evaluated the immunosuppression by these OECs and investigated the underlying mechanisms. OEC-conditioned DCs did not induce CD4 T cell polarization towards Treg, judging by the absence of FoxP3 expression. OECs also repressed T-bet/IFNγ expression in CD4 and CD8 T cells activated by DCs or anti-CD3/CD28 antibodies. This inhibition depended on OEC:T cell ratio and IFNγ repression occurred at the transcriptional level. Time-lapse experiments showed that OECs inhibited early steps of T cell activation, consistent with OECs inability to suppress T cells stimulated with PMA/ionomycin. Blocking CD40/CD40L, CD58/CD2 and PD-L1/PD-1 interactions with specific antibodies did not disrupt T cell suppression by OECs. However, preventing prostaglandin E2 (PGE2) synthesis or blocking PGE2 binding to the cognate EP2/EP4 receptors, restored IFNγ and TNFα production in OEC-conditioned T cells. Finally, treating OECs with poly(I:C), which simulates viral infections, limited T cell suppression. Overall, these results point to an inherent ability of OECs to suppress immune responses, which can nonetheless be eluded when OECs are under direct assault.

## Introduction

The oral mucosa constitutes a primary barrier for harmful pathogens, but it is also in close contact with food antigens and commensal microbiota. Therefore, there must be a fine balance between the mechanisms leading to defensive responses and immune tolerance (1). In this context, mounting evidence has demonstrated that mucosal epithelial cells have a pivotal role as initial sensors of alarm and regulators of local immune responses.

Epithelial cells in the mucosae provide a tissue-specific environment that conditions the immune response of surrounding cells such as dendritic cells (DCs) and different subsets of T cells (2, 3). For instance, in the gut mucosa, intestinal epithelial cells (IECs) constantly release retinoic acid and transforming growth factor β (TGFβ) promoting the generation of CD103+ DCs, which in turn induce the generation of regulatory T cells (Tregs) (4). Epithelial cells can also release specific cytokines such as thymic stromal lymphopoietin (TSLP), which condition DCs to promote Th2 differentiation (5, 6). In the gut mucosa, the TSLP released by IECs also conditions DCs to promote Treg differentiation (7).

Mucosal epithelial cells can also regulate T cell responses without DC mediation. They can act as antigen-presenting cells (APCs) and maintain a crosstalk with T cells (8). It has been reported that colonic, esophageal and intestinal epithelial cells express MHC II and costimulatory CD80/CD86 molecules in response to IFNγ under pathological conditions (9–12). Likewise, epithelial cells can downregulate antigen-mediated activation of alloreactive CD4 T cells, as shown in various studies using colonic, renal tubular and amniotic epithelial cells (13–15). The crosstalk between T cells and epithelial cells may also involve soluble mediators. In fact, T cells can sense many factors secreted by epithelial cells including thrombospondin, alpha-melanocyte stimulating hormone (α-MSH), TGFβ, interleukin 10 (IL-10), prostaglandin E2 (PGE2) or indoleamine 2,3-dioxygenase (IDO)-derived metabolites (16–21).

Oral epithelial cells (OECs) are also capable of modulating immune responses. In a previous work, we used OEC lines H413 and TR146 and primary OECs to show that DCs stimulated with bacteria do not fully mature in co-culture with OECs and are unable to activate alloreactive naive CD4 T cells. Similarly, OECs also suppressed the activation and response of CD4 T cells stimulated with anti-CD3/CD28 antibodies (22). In this work, we have investigated the mechanisms by which OECs suppress T cell responses and evaluated if this inhibition could be prevented. To that end, we evaluated the activation of T cells stimulated with allogenic DCs or anti-CD3/CD28 antibodies in co-culture with H413, TR146 and primary OECs. Time-lapse experiments pointed that inhibition of T cells by OECs acts on the early steps of T cell receptor (TCR) signaling, as stimulation with PMA/ionomycin prevented OEC-mediated inhibition. Interestingly, blocking PGE2 receptors on T cells and PGE2 production by OECs restored CD4 T cell activation. Also, OEC-mediated T cell suppression was limited when stimulating OECs with poly(I:C), a TLR3 agonist, emulating a viral infection. Here, we will also discuss the implications of our findings in mucosal and cancer immunology.

## Materials and Methods

### Oral Epithelial Cells (OECs)

As OECs we used two human cell lines, H413 and TR146, derived from oral squamous cell carcinomas, and primary OECs, which were collected from healthy donors after oral cavity brushing (23). Volunteers signed informed consent documents. Culture of the mentioned OECs was carried out as previously described (22). Briefly, H413 cells were grown in DMEM:HAMS F12 (1:1,vol/vol) (Gibco, NY, USA) supplemented with 0.5 μg/ml hydrocortisone sodium succinate (Merck KGaA, Darmstadt, Germany), TR146 cells in DMEM (Gibco, NY, USA), and primary OECs in RPMI 1640 (Gibco, NY, USA). All cells were grown at 37°C and 5% CO2.

### Dendritic Cells and T Cells

Monocyte-derived DCs, CD4 and CD8 T cells were obtained from peripheral blood mononuclear cells (PBMCs). PBMCs were isolated from buffy coats by a density gradient on Ficoll-Paque™ PLUS (Amersham). Buffy coats were provided by the regional blood transfusion center (Centro de Transfusion de la Comunidad de Madrid, Spain). Donors signed the informed consent document following the legislation regarding the Royal Decree-Law 1088/2005 of September 16 (BOE-A-2005-15514). Monocytes and T cells were isolated using magnetic separation kits as per the manufacturer’s instructions (CD14+ Microbeads, CD4+ T Cell Isolation Kit and CD8+ T Cell Isolation Kit; Miltenyi Biotec). DCs were obtained by differentiating monocytes as described previously (22).

### OECs Co-Cultures and Treatments

OECs were cultured in complete RPMI medium on 96-well plates (2.5 x 10^4^ cells/well) for 48 hours prior to co-culture with DCs and/or T cells. For OEC:DC co-cultures, DCs were plated alone (controls) or with OECs (1 × 10^5^ cells/well, 4 DC:1 OEC), and matured with 500 ng/mL LPS (*Escherichia coli* serotype 055:B5, Merck) for 48 h. For OEC:DC:T cell co-cultures, we added allogenic CD4 and CD8 T cells to the previous OEC:DC co-cultures (2 x 10^5^ cells/well, 8 T cells:4 DCs:1 OEC) and to the DCs alone (DC:T cell controls). DC:T cell cultures with or without OECs were incubated for 6 days, adding 10 ng/ml IL-2 (Immunotools) every 2 days.

Additionally, OECs were co-cultured with T cells alone (2 x 10^5^ T cells/well, 8 T Cells:1 OEC), previously stimulated using anti-CD3/CD28 antibodies (Dynabeads™ Human T-Activator CD3/CD28, ThermoFisher Scientific) or with 25 ng/mL PMA and 1 μg/mL ionomycin (Merck). As controls, T cells were cultured without OECs. OEC:T cell co-cultures were incubated for 4 or 48 hours depending on the experimental readout. In some experiments, OECs were pretreated before co-culture with 20 μg/mL poly(I:C) (InvivoGen) or 2 μg/mL indomethacin (Merck) for 4 h, or with 2 μg/mL anti-CD40 (HB14), anti-CD58 (TS2/9) (both from Miltenyi-Biotec) or anti-PD-L1 (MIH1) (eBioscience) for 15 min. Likewise, CD4 T cells were treated in some cases with 1 μg/mL PF-04418948 and/or ONO-AE3-208 (Merck), two selective antagonists of EP2 and EP4 receptors, respectively, for 30 min before antibody activation and co-culture with OECs. In the experiments using anti-CD40, anti-CD58, anti-PD-L1 and poly(I:C) treatments, cell cultures were washed twice with PBS and replaced with fresh media before the addition of T cells.

### Flow Cytometry

The expression of cell markers and intracellular cytokines was analyzed by flow cytometry using the following antibodies and kits: anti-T-bet (REA102), anti-IFNγ (45–15), anti-TNFα (cA2) and anti-IL-2 (JES6-5H4), from Miltenyi Biotec; anti-CD25 (BC96), anti-FoxP3 (PCH101) and anti-PD-L1 (MIH1), from eBioscience and Annexin V Apoptosis Detection Kit with 7-AAD (Biolegend). Briefly, cells were washed with 0.5% BSA 2 mM EDTA in PBS (staining buffer), Fc receptors were blocked with 200 μg/mL IgG from human serum (Merck) and cells were stained with the fluorescence-labeled antibodies. T-bet and FoxP3 were detected by intracellular staining, using a FoxP3 staining buffer set (eBioscience, San Diego, CA) following the manufacturer’s instructions. Intracellular cytokines were stained as described previously (24), incubating cells with 10 μg/mL Brefeldin A (Merck) to stop Golgi protein transport for 4 hours before the FACS staining protocol. Data were acquired using a FACSCalibur flow cytometer (BD) and analyzed using FlowJo software (Tree Star, Ashland, OR). Gating strategy is shown in **Supplementary Figure 1**.

### ELISA

Collected cell-free supernatants were analyzed using IL-6 and IL-8 Human Matched Antibody Pairs and Prostaglandin E2 Human ELISA kits (Invitrogen) as per manufacturer’s instructions. Plate readout was performed using a BioTek ELx800 Absorbance Microplate Reader.

### Quantitative RT-PCR

TR146 or CD4 T cells (>1 x 10^6^) were used for total RNA extraction (TRIzol^®^ Plus RNA Purification Kit, Invitrogen) and cDNA generation (High Capacity RNA-to-cDNA kit, ThermoFisher Scientific). *IFNB1*, *IFNG* and *CXCL10* expression was determined by RT-qPCR. The comparative method *RQ=2* ^(-ΔΔ*Ct*)^ was used to quantify mRNA transcripts and normalize IFNγ expression levels to those of *CD3E* and *IFNB1* and *CXCL10* to those of *GAPDH*. The primers used are shown in **Supplementary Table 1** and were designed with the Universal ProbeLibrary System Assay Design tool from Roche.

### Cell Proliferation Assay

OECs were seeded on 96-well plates (1.5 x 10^4^ cells/well) in complete RPMI media with or without 2 μg/mL indomethacin or 20 μg/mL poly(I:C) for 48 h. Subsequently, cell proliferation was assayed using the MTT Kit I, from Roche Diagnostics, following the manufacturer’s instructions. Absorbance was measured at 570 nm (reference 650 nm) using a BioTek ELx800 Absorbance Microplate Reader. Cell proliferation was calculated relative to the untreated control (OECs without indomethacin**).**

### Statistical Analysis

Data values were expressed as the mean ± standard error of the mean. Two-tailed Student’s *t* tests for independent samples were applied to assess statistical significance between two means and ANOVA tests for multiple comparisons between more than two means. *P* < 0.05 was considered significant. Statistic calculations were performed on GraphPad Prism 8.

## Results

### OECs Suppress T Cell Responses

**Figure 1:**
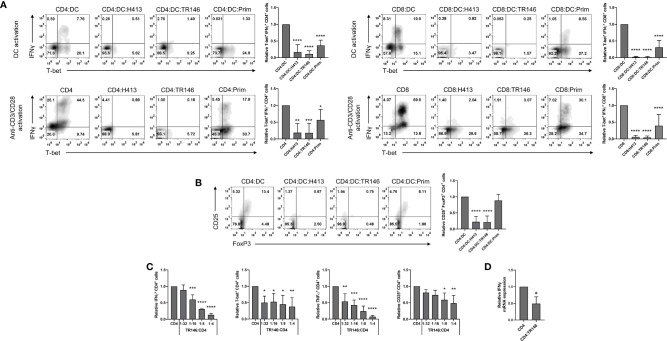
OECs restrain T cell activation and polarization. **(A)** CD4 and CD8 T cells were activated by allogenic LPS-matured DCs for 6 days (top) or with anti-CD3/CD28 antibodies for 48 h (bottom), both in the presence or not of H413, TR146 or primary (Prim.) OECs. T-bet and IFNγ expression was analyzed by flow cytometry in CD4 and CD8 T cells. **(B)** Flow cytometry analysis of CD25 and FoxP3 expression in CD4 T cells activated for 6 days by allogenic LPS-matured DCs and cultured alone or with H413, TR146 or primary (Prim) OECs. In both panels, results are shown as the percentage of CD4 T cells expressing the mentioned markers from a representative experiment (density plots) and relative to CD4 T cells alone when considering all experiments (bar graphs). **(C)** Percentage of CD4 T cells expressing T-bet, CD25, IFNγ and TNFα as determined by flow cytometry relative to CD4 T cell alone. CD4 T cells were activated by anti-CD3/CD28 beads and cultured alone or with TR146 cells for 48 hours with increasing TR146:CD4 cell number ratios. **(D)** Relative expression of *IFNG* mRNA from CD4 T cells as measured by RT-qPCR. CD4 T cells were activated by anti-CD3/CD28 beads and cultured alone or with TR146 cells for 48 hours. In all previous cases, CD4 T cells were re-stimulated with PMA and ionomycin for 4 hours before analysis. FACS gatings were adjusted by the use of mouse anti-IgG-PE and anti-IgG-APC antibodies. We plotted mean values with error bars corresponding to SEM. Statistically significant differences were obtained applying ANOVA tests **(A-C)** or two-tailed Student’s *t* tests for independent samples **(D)**. Significant differences (p < 0.05, p < 0.01, p < 0.001 or p < 0.0001) are noted as “*”, “**”, “***”, and “****”, respectively. Data were obtained from a total of three independent experiments using samples from different donors.

We previously reported that OECs suppress DC-mediated activation of CD4 T cells, abrogating IFNγ and TNFα release as determined by ELISA (22). Here, we evaluated in more detail this immunosuppression in both CD4 and CD8 T cells using intracellular staining and FACS analysis. To that end, allogenic CD4 and CD8 T cells were cultured with LPS-matured DCs alone (control) or OEC-conditioned DCs for 6 days. Alternatively, anti-CD3/CD28 antibodies were used instead of DCs to stimulate CD4 and CD8 T cells with and without (controls) OECs. As OECs we used H413, TR146 and primary OECs. To examine the activation of effector Th1 cells, we analyzed the expression of T-bet and IFNγ. Stimulation of CD4 and CD8 T cells with allogenic DCs or anti-CD3/CD28 antibodies resulted in large amounts of T-bet+ IFNγ-producing cells (**Figure 1A**). In contrast, the presence of OECs prevented the expansion and/or differentiation of T-bet+ T cells and their activation, as judge by the IFNγ production. Similarly, the population of CD25+FoxP3+ CD4 T cells observed in the cultures of CD4 T cells stimulated with DC decreased drastically in the presence of OECs (**Figure 1B**). In both cases, primary OECs showed less immunosuppressive effect on T cells than H413 or TR146 cells, possibly due to their reduced viability.

To analyze if the observed inhibition by OECs depended on the OEC:CD4 ratio we activated CD4 T cells with anti-CD3/CD28 antibodies and co-cultured them for 48 hours with increasing numbers of TR146 cells, as representative OECs. We then determined CD4 T cells expression levels of CD25, T-bet, IFNγ and TNFα. We observed that the expression of these molecules decreased as the TR146:CD4 ratio increased, indicating a dose-dependent inhibitory effect (**Figure 1C**). To determine if IFNγ production was inhibited at the transcriptional or translational level, we examined *IFNG* mRNA expression by quantitative RT-PCR in anti-CD3/CD28-activated CD4 T cells incubated or not with TR146 cells. As observed in **Figure 1D**, *IFNG* transcription in CD4 T cells was strongly reduced in the presence of TR146 cells (>50%) compared to control activated T cells. This strong reduction in *IFNG* transcription levels explains the marginal production of this cytokine by T cells in the presence of OECs.

### OEC-Dependent Inhibition of T Cells Targets Early TCR Signaling Events

**Figure 2:**
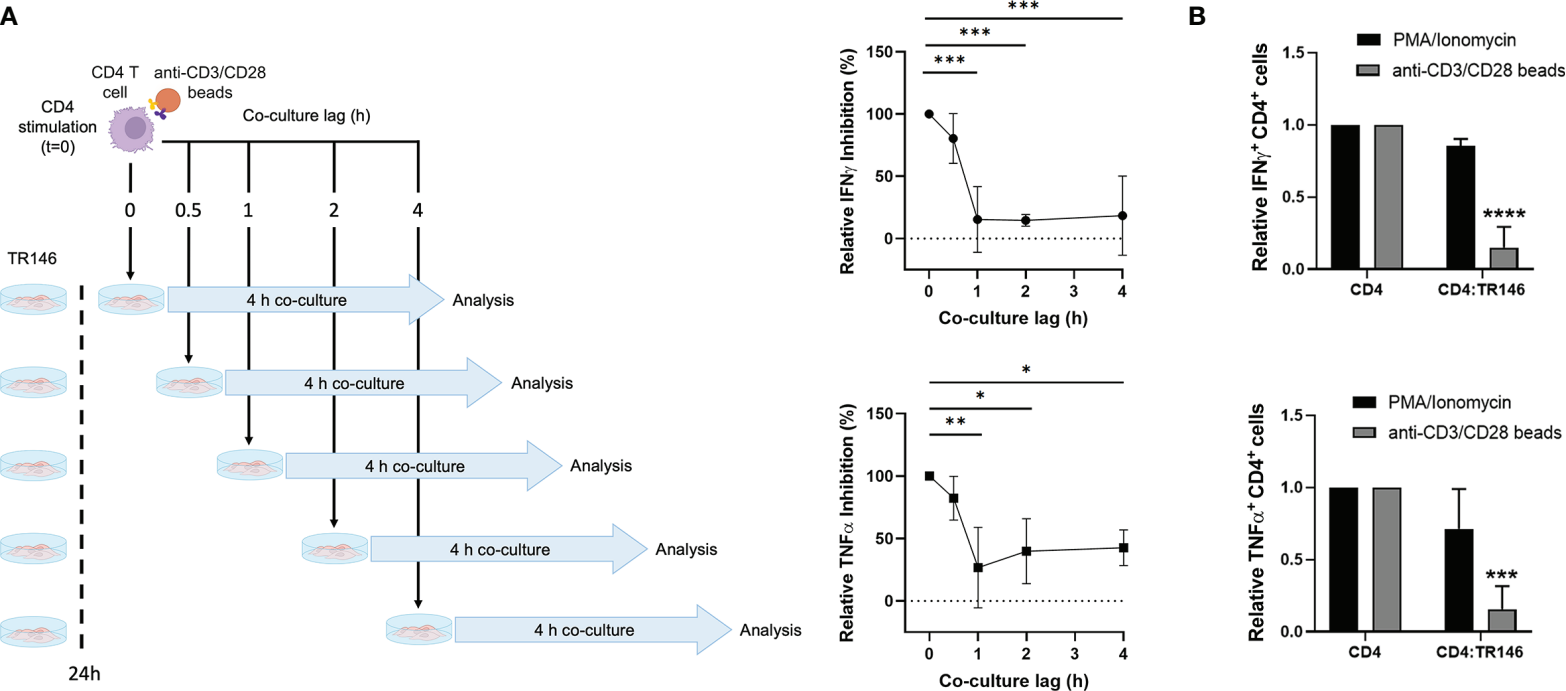
CD4 T cell inhibition by TR146 cells takes place early during TCR signaling. **(A)** CD4 T cells were incubated with anti-CD3/CD28 antibodies for different times (0, 0.5, 1 or 2 hours) before an additional 4-hour co-culture with TR146 cells. Activated CD4 T cells were also cultured alone. Schematic diagram on the left depicts the experiment workflow. The percentage of CD4 T cells that were inhibited when cultured with TR146 cells in comparison to CD4 T cells cultured alone for each co-culture lag time were detected by flow cytometry. Data are expressed as the percentage of IFNγ and TNFα inhibition relative to control of maximum inhibition (co-culture lag time = 0 h). **(B)** CD4 T cells were activated with PMA/ionomycin or anti-CD3/CD28 beads and cultured alone or with TR146 cells for 4 hours. The amount of IFNγ and TNFα-producing CD4 T cells was measured by flow cytometry and expressed as relative to control. FACS gatings were adjusted by the use of mouse anti-IgG-PE and anti-IgG-APC antibodies. All data are plotted as mean values with error bars corresponding to SEM. Statistically significant differences were obtained applying ANOVA tests. Significant differences (p < 0.05, p < 0.01, p < 0.001 and p < 0.0001) are noted as “*”, “**”, “***” and “****”, respectively. Data were obtained from a total of three independent experiments using samples from different donors.

To gain further insight into the dynamics of the inhibition by OECs, we performed time-lapse experiments by delaying the co-culture of activated T cells with OECs. CD4 T cells were stimulated with anti-CD3/CD28 antibodies for 0, 0.5, 1 and 2 h prior to co-culture with TR146 cells, as illustrated in **Figure 2A** (left side). After a 4-hour co-culture, we monitored the percentage of IFNγ and TNFα-producing T cells (**Figure 2A**). When CD4 T cells were co-cultured with OECs immediately after anti-CD3/CD28 stimulation (co-culture lag = 0 hours), IFNγ and TNFα-producing T cell populations were completely abrogated in relation to control CD4 T cells. However, when T cells were stimulated for 1 hour or longer prior to TR146 co-culture, the percentage of T cell inhibition dropped to only 15-30% of that of T cells co-cultured immediately after anti-CD3/CD28 stimulation. This result suggests that the inhibitory mechanism(s) mediated by OECs operated promptly during T cell activation. We also used phorbol 12-myristate 13-acetate (PMA), that directly activates Protein Kinase C (PKC) (25), and ionomycin, a calcium ionophore that increases cytoplasmic Ca^2+^ concentration (26) to activate CD4 T cells before co-culture with OECs. The activation of T cells by these two compounds bypasses the first events of T cell receptor (TCR) signaling. We observed that, opposite to anti-CD3/CD28 stimulation, TR146 cells were unable to inhibit IFNγ and TNFα production by T cells activated by PMA and ionomycin (**Figure 2B**), supporting that OECs inhibition acts on early steps of TCR signaling, preceding PKC activation and calcium release from the endoplasmic reticulum.

### T Cell Suppression Is Independent of CD40, CD58 and PD-L1 Expression by OECs

**Figure 3:**
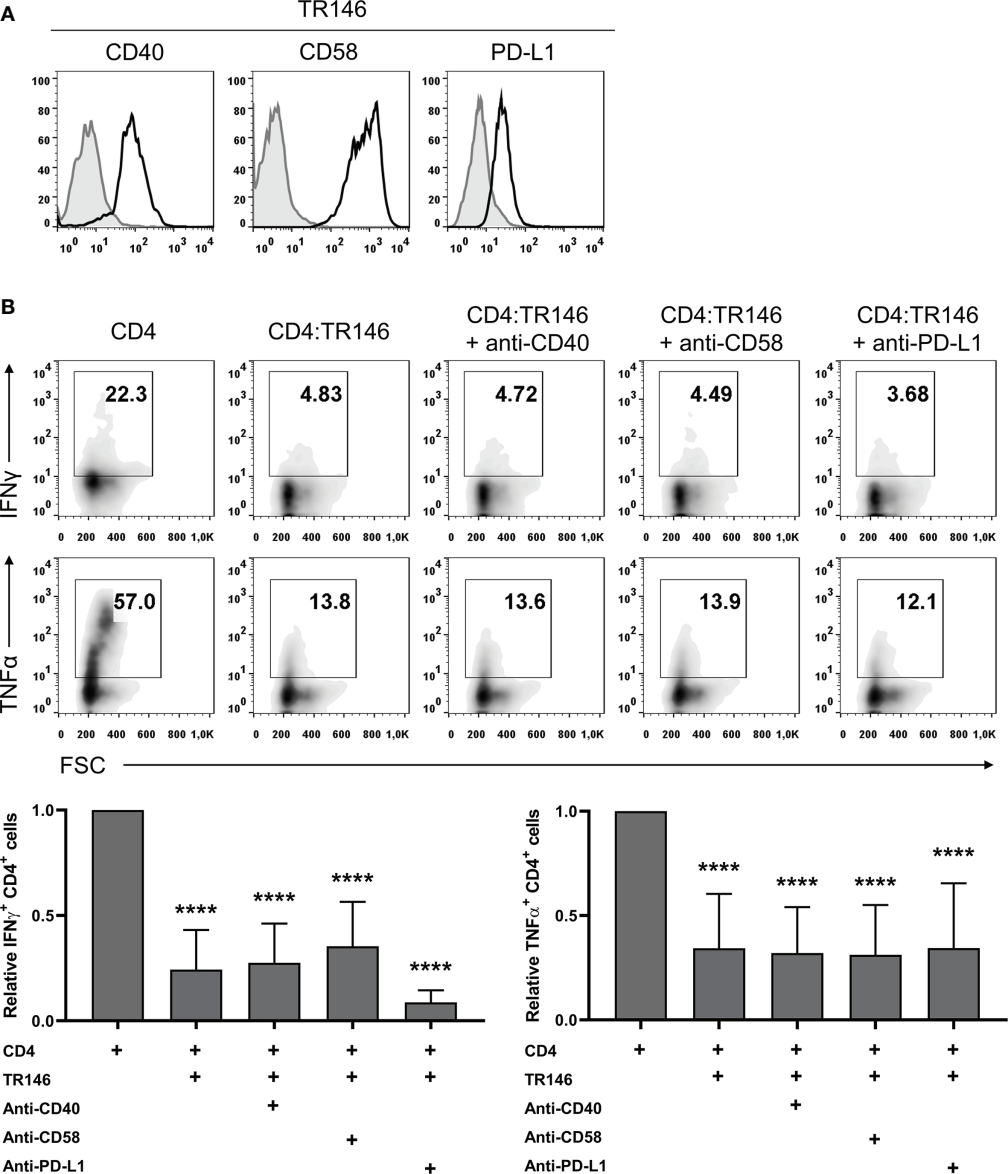
CD40, CD58 or PD-L1 in TR146 cells do not participate in CD4 T cells inhibition. **(A)** TR146 cells surface expression of CD40, CD58 and PD-L1 (black) compared with isotype control (grey), as detected by flow cytometry. **(B)** TR146 cells were incubated with anti-CD40, anti-CD58 or anti-PD-L1 blocking antibodies for 15 min and cultured with CD3/CD28-activated CD4 T cells for 4 hours. Activated CD4 T cells were cultured alone as a control. Data were collected by flow cytometry and shown as the percentage of IFNγ and TNFα-producing CD4 T cells from a representative experiment (density plots) and relative to CD4 T cells alone when considering all experiments (bar graphs). FACS gatings were adjusted by the use of mouse anti-IgG-PE and anti-IgG-APC antibodies. Bar graphs display mean values with SEM error bars. Statistically significant differences were obtained applying ANOVA tests. Significant differences (p < 0.001) are noted as “****”. Data were obtained from a total of four independent experiments using samples from different donors.

Epithelial cells can express various surface molecules that allow them to engage T cells and modulate their response (27–29). Here, we evaluated the potential role of three of these molecules, CD40, CD58 and PD-L1, in the observed immunosuppression, using TR146 cells as representative OECs. We first confirmed that TR146 cells express detectable levels of these three proteins (**Figure 3A**). Subsequently, we incubated OECs with anti-CD40, anti-CD58 and anti-PD-L1 blocking antibodies for 15 min, co-cultured them with anti-CD3/CD28-activated CD4 T cells and analyzed cytokine production. As shown in **Figure 3B**, these antibodies were unable to prevent the inhibition by OECs.

### OEC Suppression of T Cell Responses Is Largely Mediated by PGE2

**Figure 4:**
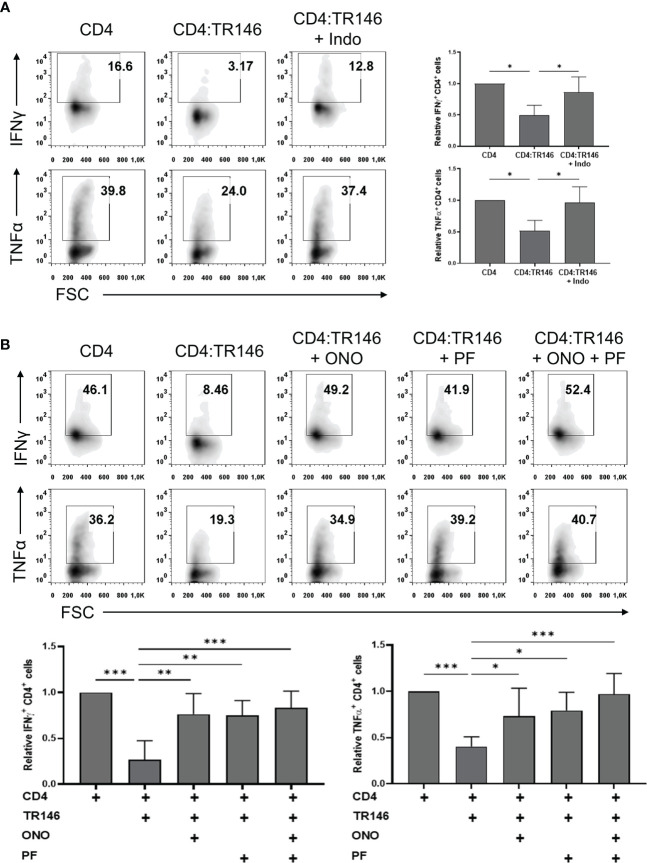
TR146 cells suppress CD4 T cells *via* PGE2 release. **(A)** TR146 cells were treated or not with 2 μg/mL indomethacin (Indo) for 4 hours and co-cultured with anti-CD3/CD28-activated CD4 T cells for 4 hours. **(B)** CD4 T cells were treated with PF-04418948 (PF) and/or ONO-AE3-208 (ONO) inhibitors for 1 hour, activated with anti-CD3/CD28 beads and co-cultured with TR146 cells for 4 hours. All data were obtained by flow cytometry analysis and shown as the percentage of IFNγ and TNFα-producing CD4 T cells from representative experiments (density plots) and relative to CD4 T cells alone when considering all experiments (bar graphs). FACS gatings were adjusted by the use of mouse anti-IgG-PE and anti-IgG-APC antibodies. Bar graphs display mean values with SEM error bars. Statistically significant differences were obtained applying ANOVA tests. Significant differences (p < 0.05, p < 0.01 and p < 0.001) are noted as “*”, “**” and “***”, respectively. Data were obtained from a total of four independent experiments using samples from different donors.

Mounting evidence supports that prostaglandin E2 (PGE2) can act as an anti-inflammatory mediator by suppressing immune responses (20, 30). To examine if OECs suppress T cell responses by releasing PGE2 we used indomethacin, an inhibitor of COX-1 and COX-2 enzymes involved in PGE2 synthesis (31). PGE2 production by TR146 cells (about 5000 pg/mL), was significantly abrogated following indomethacin treatment, although it did not affect cell viability and proliferation (**Supplementary Figure 2**). PGE2 levels in H413 cell cultures were similar to those found in TR146 cell cultures and around 100 pg/mL in primary OECs cultures, and could all be downregulated by indomethacin (data not shown). TR146 cells treated with or without indomethacin were co-cultured with anti-CD3/CD28-activated CD4 T cells and cytokine production was monitored. As shown earlier, the production of IFNγ and TNFα by T cells was inhibited by OECs. However, this inhibition was prevented when TR146 cells were previously treated with indomethacin (**Figure 4A**). In this situation, IFNγ and TNFα-producing T cells in TR146 co-cultures were comparable to those in activated control T cells. The concentration of indomethacin used in these assays, 2 µg/mL, was selected after dose-response experiments (**Supplementary Figure 3A**). COX-1/COX-2 enzymes mediate the synthesis of more prostanoids than just PGE2 (32). To verify that PGE2 is indeed involved in T cell inhibition, we incubated CD4 T cells with PF-04418948 and ONO-AE3-208, which block PGE2 binding to EP2 and EP4, respectively (33, 34), prior to CD3/CD28 stimulations and co-culture with OECs. Consistent with the results of indomethacin treatment, PF-04418948 and ONO-AE3-208 restored 75-100% of IFNγ and TNFα-producing T cells in TR146 co-cultures, confirming that PGE2 released by OECs mediated T cell inhibition (**Figure 4B**). Simultaneous incubation with both receptor antagonists only increased T cell cytokine production marginally, compared to T cells treated with the antagonists individually. We also carried out dose-response experiments with PF-04418948 and/or ONO-AE3-208, finding that 1 μg/mL of these compounds was enough to prevent OECs-mediated immunosuppression (**Supplementary Figure 3B**). Similar results were found in T cells co-cultured with H413 cells and primary OECs, either treating OECs with indomethacin or T cells with PF-04418948 and/or ONO-AE3-208, with the exception of primary OECs, which did not inhibit TNFα in any case (**Supplementary Figure 4**). Treatment of CD4 T cells with PF-04418948 and/or ONO-AE3-208 alone did not alter IFNγ and TNFα production in comparison to untreated CD4 T cells (**Supplementary Figure 5**).

### OECs Exposed to a Viral-Like Insult Permit Partial T Cell Responses

**Figure 5:**
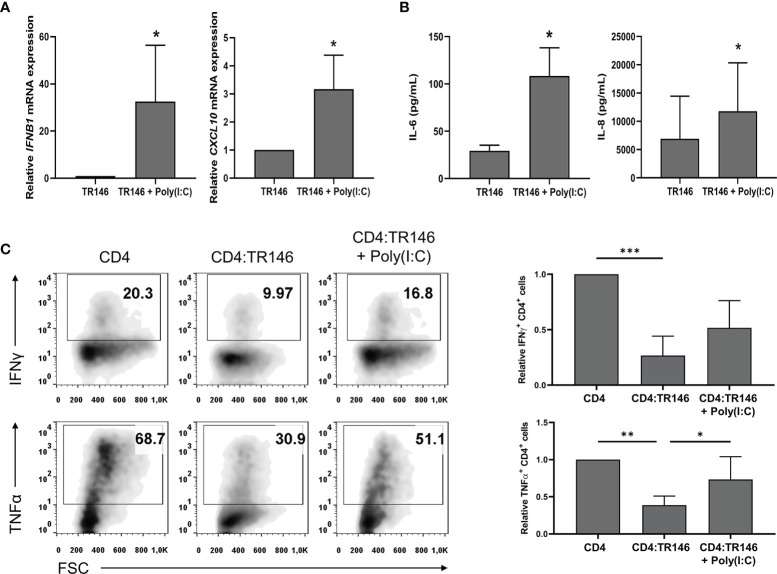
Poly(I:C) stimulates cytokine expression in TR146 cells and partially prevents T cell inhibition by OECs. TR146 cells were first stimulated or not with 20 μg/mL poly(I:C) for 4 hours and then washed twice with PBS. **(A)** *IFNB1* and *CXCL10* mRNA relative expression was evaluated in TR146 cells by RT-qPCR. **(B)** TR146 cells were cultured for other 48 hours and IL-6 and IL-8 release was measured by ELISA in cell-free supernatants. **(C)** TR146 cells were co-cultured with anti-CD3/CD28-activated CD4 T cells for other 48 hours and re-stimulated with PMA and ionomycin for 4 hours before analysis. Data were obtained by flow cytometry and shown as the percentage of IFNγ and TNFα-producing CD4 T cells from a representative experiment (density plots) and relative to CD4 T cells alone when considering all experiments (bar graphs). FACS gatings were adjusted by the use of mouse anti-IgG-PE and anti-IgG-APC antibodies. Bar graphs display mean values with SEM error bars. Statistically significant differences were obtained applying two-tailed Student’s *t* tests for independent samples **(A, B)** or ANOVA tests **(C)**. Significant differences (p < 0.05, p < 0,01 and p < 0.001) are noted as “*”, “**” and “***”, respectively. Data were obtained from a total of three independent experiments using samples from different donors.

To emulate OECs behavior under viral infections, we challenged TR146 cells with poly(I:C), a synthetic analog of viral dsRNA that activates TLR3. As shown in **Figures 5A, B**, poly(I:C) stimulated the expression of *IFNB1* and *CXCL10* and the release of IL-6 and IL-8 in TR146 cells. In addition, poly(I:C)-treated TR146 cells could not completely suppress the response of anti-CD3/CD28-activated CD4 T cells; there were about twice more IFNγ and TNFα-producing CD4 T cells in co-cultures with treated OECs than in co-cultures with non-treated OECs (**Figure 5C**). The amount of poly(I:C) used to treat OECs (20 μg/mL) was selected upon dose-response assays (**Supplementary Figure 3C**). Interestingly, PGE2 levels were slightly increased in poly(I:C)-treated TR146 cell cultures (**Supplementary Figure 2A**).

## Discussion

Epithelial cells are fundamental for the regulation of mucosal immunity and those in the oral mucosa are not an exception (7, 35). We have previously shown that, in co-cultures, OECs suppress DC and T cell responses to various stimuli, including bacteria. In this context, we concluded that oral epithelial cells mandate an immune quiescence that prevents undesired immune responses (22). Here, we have further investigated the inhibition of T cells by OECs. To that end, we used the OEC lines H413 and TR146, and primary OECs in co-culture with activated T cells, either with allogenic DCs or with anti-CD3/CD28 antibodies. For many mechanistic/blocking studies, we chose TR146 cells as the representative OEC line since they grow and proliferate better than H413 cells. Moreover, PD-L1 cell surface expression in TR146 cells is higher than in H413 cells and T cell inhibition through the PD-1/PD-L1 axis, if any, could be better detected using TR146 cells.

**Figure 6:**
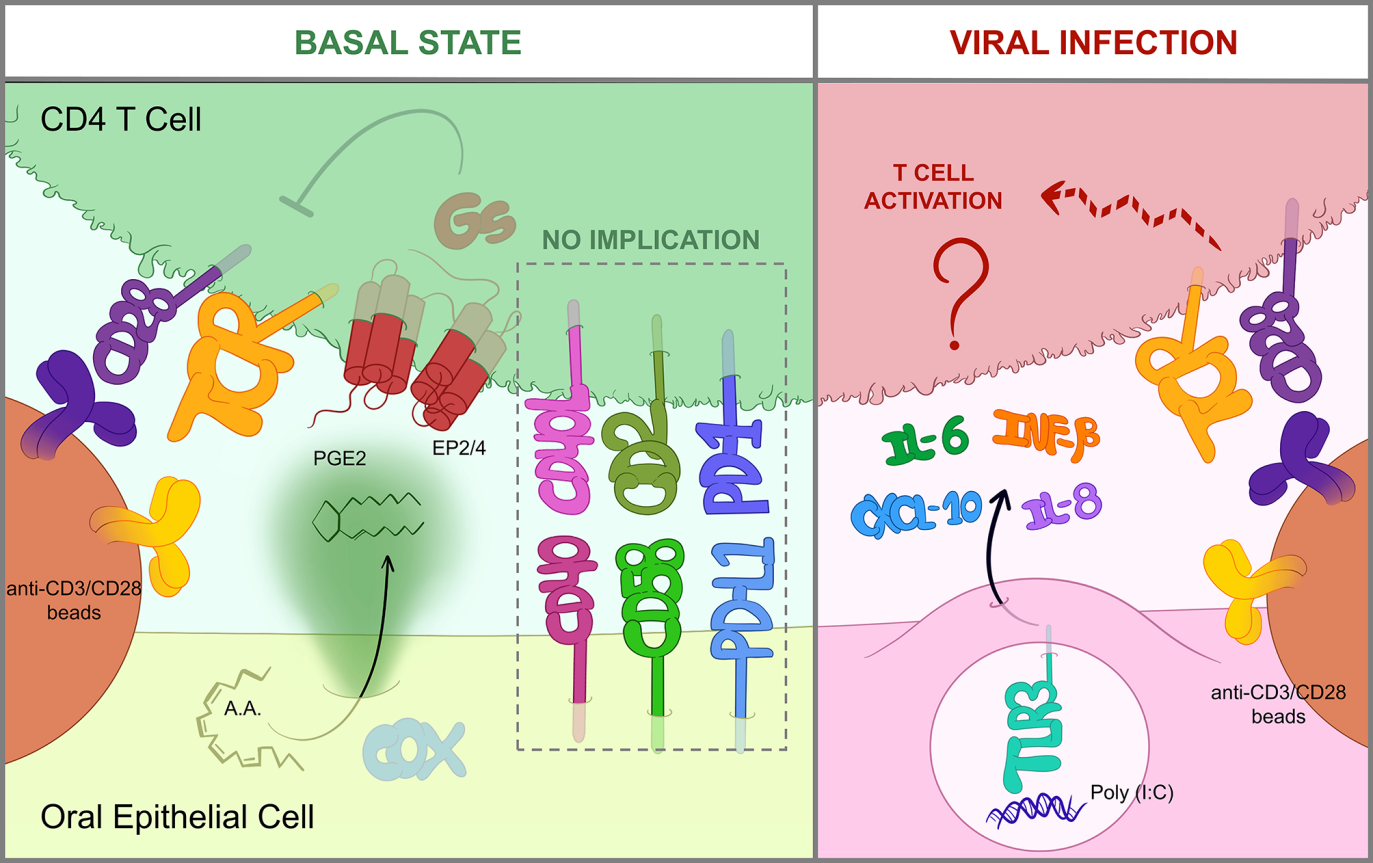
OEC-mediated immunomodulation of T cells. Graphical summary of the most relevant findings obtained in this study. In a basal state (left), OECs constitutively suppress T cells through the action of PGE2, which is produced by COX enzymes from arachidonic acid (AA) and then sensed by EP2/EP4 receptors in the T cell. EP2/EP4 signaling occur through Gs-proteins and eventually blocks TCR signaling, thus hindering activation by anti-CD3/CD28 antibodies (36). Cell-to-cell contacts including CD40/CD40L, CD58/CD2 or PD-L1/PD-1 are not involved in OEC-derived inhibition. Contrarily, when OECs are exposed to viral insults like poly(I:C), a dsRNA mimic and TLR3-inducer, they express IL-6, IL-8, *IFNB1* and *CXCL10* and relieve CD4 T cell suppression by an unknown mechanism (right).

The most relevant findings of the study are summarized in **Figure 6**. We demonstrated that impairing PGE2 synthesis or its binding to cognate receptors in T cells prevented the inhibition of CD4 T cell responses by OECs. Treating OECs with indomethacin, a non-selective inhibitor of COX enzymes, or T cells with specific antagonists of PGE2 receptors EP2 and EP4 (PF-04418948 and ONO-AE3-208, respectively) allowed T cell responses, judging by IFNγ and TNFα productions. PGE2 is a soluble mediator synthesized by the constitutive COX-1 and the inducible COX-2 enzymes, which can inhibit the TCR signaling cascade in T cells (37). *In vitro*, several studies have already shown that the addition of PGE2 to T cell cultures at concentrations in the range of 0.1 nM to 10 μM inhibits T cell activation/responses (37–39). EP2 and EP4 receptors promote cAMP production and PKA activation leading to the phosphorylation of the C-terminal Src kinase (Csk), which eventually interrupts the TCR signaling cascade by inactivating Lck (36, 40). In line with this, when we activated T cells with PMA and ionomycin, targeting signaling events downstream of Lck activation, OECs suppression was prevented, as this activation eludes the inhibitory mechanism of PGE2. The ability of epithelial cells, including OECs, to secrete this prostanoid has been previously reported (41, 42). PGE2 is present in human saliva at a concentration of around 0.1 nM (43) comparable to what we found in primary OECs cultures (0.3 nM, data not shown), enough to inhibit T cell activation. The immunoregulatory role of PGE2 has been widely studied in different mucosae. PGE2 is essential for the homeostasis of the gastrointestinal tract (20, 44, 45) and has emerged as a local protection factor in a number of epithelia, like retinal, bronchial, glomerular or biliary epithelia (42, 46–48). Interestingly, tumoral epithelial cells synthesize huge amounts of PGE2 in order to create a suppressive environment (49). It is worth noting that 80 to 90% of all cancer cases are caused by epithelial malignancies^1^. In this context, indomethacin has been reported to have an anti-tumoral potential, which has been attributed to its capacity to inhibit cellular calcium mobilization (50) and angiogenesis through VGEF downregulation (51). However, an alternative mechanism suggested by our study will be that indomethacin facilitates tumor specific T cell responses by precluding the release of PGE2.

The observed effect of OECs on T cells was dose-dependent and immediate, operating faster than CD3/CD28 stimulation. PGE2 secretion enabled OECs to inhibit T cell responses and likely also their differentiation. A similar phenomenon has been observed in the intestinal mucosa, where epithelial cells reduce the mRNA levels of *IL2*, *IFNG*, *IL4* and *IL5* in CD4 T cells (52). In our experiments, the inhibition of T cell activation was not explained by an enhancement of CD25+FoxP3+ Treg cells. In the absence of epithelial cells, some authors have reported that PGE2 inhibited T-bet and IFNγ expression in CD4 T cells but promoted their differentiation to Treg (53) or Th17 cells (54). *In vivo*, there is evidence that the lymphoid tissue associated with the oral mucosa is highly enriched in Treg cells (55), but the origin of these cells is yet unclear. According to Tanaka et al. (56), murine oral classical DCs induced antigen-specific FoxP3+ Treg cells in draining submandibular lymph nodes but not directly in the oral mucosa. Therefore, while the PGE2 produced by OECs is probably sufficient to perform T cell suppression *in vitro*, the lack of a microenvironmental context may be impeding Treg promotion. Taken together, and compatible with Treg-derived immunomodulation, our data support that PGE2 secretion by oral epithelial cells acts as an innate and default immunosuppressive mechanism that prevent undesired responses. Moreover, it is also tantalizing to speculate that the elicitation of T cell responses in secondary lymphoid tissues with the additional need for antigen delivery/transport has evolved as a mechanism to elude the immunosuppressive effect of epithelial cells.

We previously hypothesized that immune suppression by OECs was contact-dependent as OECs-conditioned media did not have much effect on T cells (22). Others have also shown that epithelial cells inhibit T cell responses through cell-to-cell contacts. For instance, tubular renal and iris pigment epithelial cells express PD-L1 and suppress T cell activation in this way (57, 58). We also found that OECs expressed PD-L1 as well as other surface markers, including CD58 and CD40, which are implicated in T cell adhesion and co-stimulation (13, 59–63). However, none of these proteins mediated the observed inhibition in our experimental settings. Moreover, we found that PGE2 secretion by OECs is a main mechanism implicated in T cell suppression. This result could appear paradoxical. Nevertheless, we cannot rule out that other cell-contact mechanisms can participate in the inhibition of T cells. Moreover, the action of PGE2 is likely enhanced by the proximity between OEC and T cells. In fact, it also worth noting that PGE2 is a highly hydrophobic molecule and probably remains bound to membranes close to the secretion sites.

PGE2 release by OECs is constitutive (43), representing a constant immunosuppressive mechanism that has presumably evolved to tolerate the presence of resident bacteria. However, this default immunosuppression should be eluded under some threatening conditions. In fact, we found that OECs treated with poly(I:C), a TLR3 agonist simulating a viral infection, did not completely prevent T cell activation. In line with these results, Schwarze et al. (64) showed that poly(I:C) can attenuate T cell suppression by airway epithelial cells. Furthermore, *in vivo* poly(I:C) treatment of epithelial carcinomas has also been shown to promote T cell tumor infiltration and responses (65, 66). The mechanism by which OECs treated with poly(I:C) had a limited ability to inhibit T cells did not involve downregulation of PGE2 production. OECs treated with poly(I:C) exhibit enhanced expression of *IFNB1* and *CXCL10* and release of IL-6 and IL-8. However, the addition of IFNβ1, CXCL10, IL-6 and IL-8 cytokines in OECs and T cell co-cultures was not sufficient to reproduce the effect of poly(I:C) treatment (data not shown), suggesting the involvement of other unknown mediators capable of counteracting the inhibitory effect of PGE2. In summary, our results indicate that PGE2 release by OECs contributes to restrain T cells and maintain a tolerant environment. However, under certain conditions like a viral assault, OECs can lift T cell immunosuppression through a PGE2-independent mechanism that may involve the expression of yet to discover contact-dependent or soluble factors.

## Data Availability Statement

The original contributions presented in the study are included in the article/**Supplementary Material**. Further inquiries can be directed to the corresponding authors.

## Ethics Statement

The studies involving human participants were reviewed and approved by CEIm from Hospital Clínico San Carlos, Madrid, Spain. The patients/participants provided their written informed consent to participate in this study.

## Author Contributions

EML and PAR: conceptualization. JS-T and HP-P: methodology. JS-T, HP-P, EL, and PAR: investigation. JS-T, HP-P, EML and PAR: writing-original draft. JS-T, EML, and PAR: final writing and editing. All authors contributed to the article and approved the submitted version.

## Conflict of Interest

The authors declare that the research was conducted in the absence of any commercial or financial relationships that could be construed as a potential conflict of interest.

## Publisher’s Note

All claims expressed in this article are solely those of the authors and do not necessarily represent those of their affiliated organizations, or those of the publisher, the editors and the reviewers. Any product that may be evaluated in this article, or claim that may be made by its manufacturer, is not guaranteed or endorsed by the publisher.

## Footnotes

1 SEER Training Modules, Cancer Classification. U. S. National Institutes of Health, National Cancer Institute. https://training.seer.cancer.gov/disease/categories/classification.html [Accessed May 11 2021].

## Supplementary Material

Supplementary Table 1 List of primers used for real-time PCR analysis.

Supplementary Figure 1 Gating strategy for T cell flow cytometry analysis. Isolated CD4 T cells were stained with the antibodies of interest or their isotype controls (mouse anti-IgG antibodies) and analyzed by flow cytometry. In both cases the antibodies were conjugated to FITC, PE or APC fluorochromes. Gating of the CD4 T cell population was determined based on SSC and FSC parameters. On this population the signal from isotype controls was used to define the ground fluorescence produced by stimulated CD4 T cells and adjust the positive region for FITC, PE or APC fluorescence emission. This gating conditions were used to analyze flow cytometry data. As an example, we have shown the gating strategy for detecting IFNγ+ (APC) and TNFα+ (PE) double positive cells (upper plots, APC vs PE) or IFNγ+ cells (lower plots, APC vs FSC).

Supplementary Figure 2 Effect of indomethacin and poly(I:C) treatment on TR146 cells. TR146 cells were treated with 2 μg/mL indomethacin (Indo) or 20 μg/mL poly(I:C) for 48 hours. **(A)** PGE2 release was measured by ELISA in cell-free supernatants. **(B)** Cell proliferation was measured by MTT assay (left) and viability was analyzed by flow cytometry, staining cells with 7-AAD and Annexin V, as shown in density plots from a representative experiment (right). FACS gates were adjusted by the use of a mouse anti-IgG-FITC antibody (for Annexin V) and unstained cells for 7-AAD. Bar graphs display mean values with SEM error bars. Statistically significant differences (p < 0.05) were noted as “*”. Data were obtained from a total of three independent experiments using samples from different donors.

Supplementary Figure 3 Dose-response effect of indomethacin, EP2/EP4 receptors antagonists and poly(I:C). **(A)** TR146 cells were treated or not with different concentrations of indomethacin (Indo) (0.01, 0.5 and 2 μg/mL) for 4 hours and co-cultured with anti-CD3/CD28-activated CD4 T cells for 4 hours. **(B)** CD4 T cells were treated with different concentrations of PF-04418948 (PF) and/or ONO-AE3-208 (ONO) inhibitors (0.05, 0.1 and 1 μg/mL) for 1 hour, activated with anti-CD3/CD28 beads and co-cultured with TR146 cells for 4 hours. **(C)** TR146 cells were stimulated or not with different concentrations of poly(I:C) (1, 5 and 20 μg/mL) for 4 hours and then washed twice with PBS. TR146 cells were co-cultured with anti-CD3/CD28-activated CD4 T cells for other 48 hours and restimulated with PMA and ionomycin for 4 hours before analysis. All data were obtained by flow cytometry analysis and shown as the percentage of IFNγ (upper plots) and TNFα-producing (lower plots) CD4 T cells from a representative experiment. FACS gates were adjusted by the use of mouse anti-IgG-PE and anti-IgG-APC antibodies.

Supplementary Figure 4 H413 cells and primary OECs suppress CD4 T cells through PGE2 production. **(A)** H413 cells and **(B)** primary OECs were treated or not with 2 μg/mL indomethacin (Indo) for 4 hours and co-cultured with anti-CD3/CD28-activated CD4 T cells for 4 hours. **(C)** H413 cells or **(D)** primary OECs were cocultured 4 hours with anti-CD3/CD28-activated CD4 T pretreated for 1 hour with PF-04418948 (PF) and/or ONO-AE3-208 (ONO) inhibitors. All data were collected by flow cytometry and shown as the amount of IFNγ and TNFα-producing CD4 T cells relative to CD4 T cells alone. FACS gatings were adjusted by the use of mouse anti-IgG-PE and anti-IgG-APC antibodies. Bar graphs display mean values with SEM error bars. Statistically significant differences (p < 0.05, p < 0,01 and p < 0.001) are noted as “*”, “**” and “***”, respectively. Data were obtained from a total of three independent experiments using samples from different donors.

Supplementary Figure 5 Treatment of CD4 T cells with PF-04418948 and ONO-AE3-208 inhibitors do not alter IFNγ and TNFα production. CD4 T cells were treated with PF-04418948 (PF) and/or ONO-AE3-208 (ONO) inhibitors for 1 hour, activated with anti-CD3/CD28 beads and cultured alone for 4 hours. Data were collected by flow cytometry and shown as the amount of IFNγ and TNFα-producing CD4 T cells relative to untreated CD4 T cells. FACS gatings were adjusted by the use of mouse anti-IgG-PE and anti-IgG-APC antibodies. Bar graphs display mean values with SEM error bars. Data were obtained from a total of three independent experiments using samples from different donors.
