# Supplementary material for: Human Oral Epithelial Cells Suppress T Cell Function via Prostaglandin E2 Secretion
Source: Front Immunol. 2022 Jan 19;12:740613. doi: 10.3389/fimmu.2021.740613 (PMC8807503; doi:10.3389/fimmu.2021.740613)
Supplement: Supplementary Table 1 — List of primers used for real-time PCR analysis. [file Table_1.docx]

**Supplementary Table 1.** List of primers used for real-time PCR analysis

| **Gene** | **Forward primer** | **Reverse primer** |
| --- | --- | --- |
| *IFNB1* | 5’-CTTTGCTATTTTCAGACAAGATTCA-3’ | 5’-GCCAGGAGGTTCTCAACAAT-3’ |
| *IFNG* | 5’-GGCATTTTGAAGAATTGGAAAG-3’ | 5’-TTTGGATGCTCTGGTCATCTT-3’ |
| *CXCL10* | 5’-GAAAGCAGTTAGCAAGGAAAGGT-3’ | 5’-GACATATACTCCATGTAGGGAAGTGA-3’ |
| *CD3E* | 5’-GCACTCACTGGAGAGTTCTGG-3’ | 5’-CCACCCATTTCTTCATTACCA-3’ |
| *GAPDH* | 5’-CCCCGGTTTCTATAAATTGAGC-3’ | 5’-CTTCCCCATGGTGTCTGAG-3’ |
