# Supplementary figures and images for: Human Oral Epithelial Cells Suppress T Cell Function via Prostaglandin E2 Secretion
Source: Front Immunol. 2022 Jan 19;12:740613. doi: 10.3389/fimmu.2021.740613 (PMC8807503; doi:10.3389/fimmu.2021.740613)

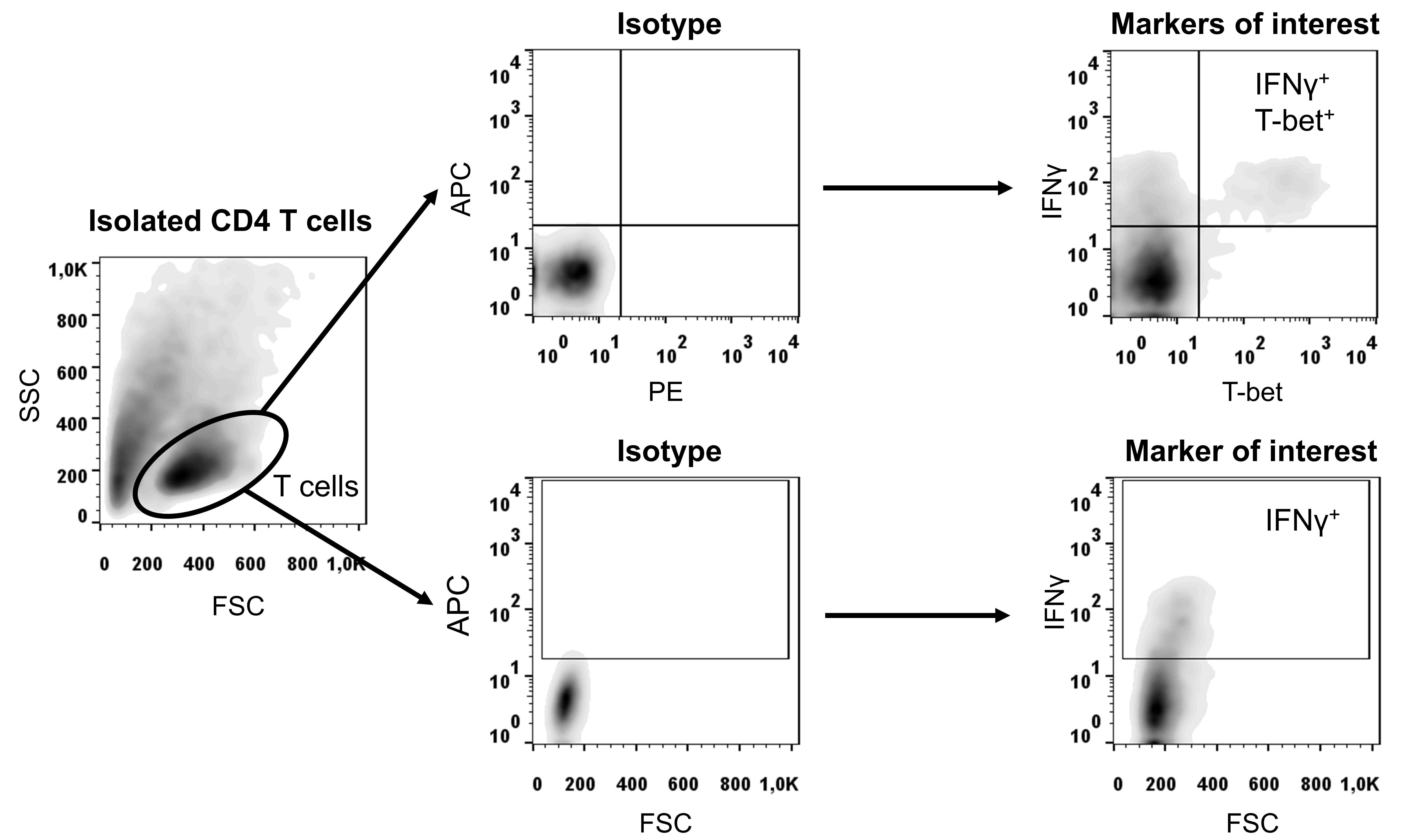

Supplement: Supplementary Figure 1 — Gating strategy for T cell flow cytometry analysis. Isolated CD4 T cells were stained with the antibodies of interest or their isotype controls (mouse anti-IgG antibodies) and analyzed by flow cytometry. In both cases the antibodies were conjugated to FITC, PE or APC fluorochromes. Gating of the CD4 T cell population was determined based on SSC and FSC parameters. On this population the signal from isotype controls was used to define the ground fluorescence produced by stimulated CD4 T cells and adjust the positive region for FITC, PE or APC fluorescence emission. This gating conditions were used to analyze flow cytometry data. As an example, we have shown the gating strategy for detecting IFNγ+ (APC) and TNFα+ (PE) double positive cells (upper plots, APC vs PE) or IFNγ+ cells (lower plots, APC vs FSC). [file Image_1.jpeg]

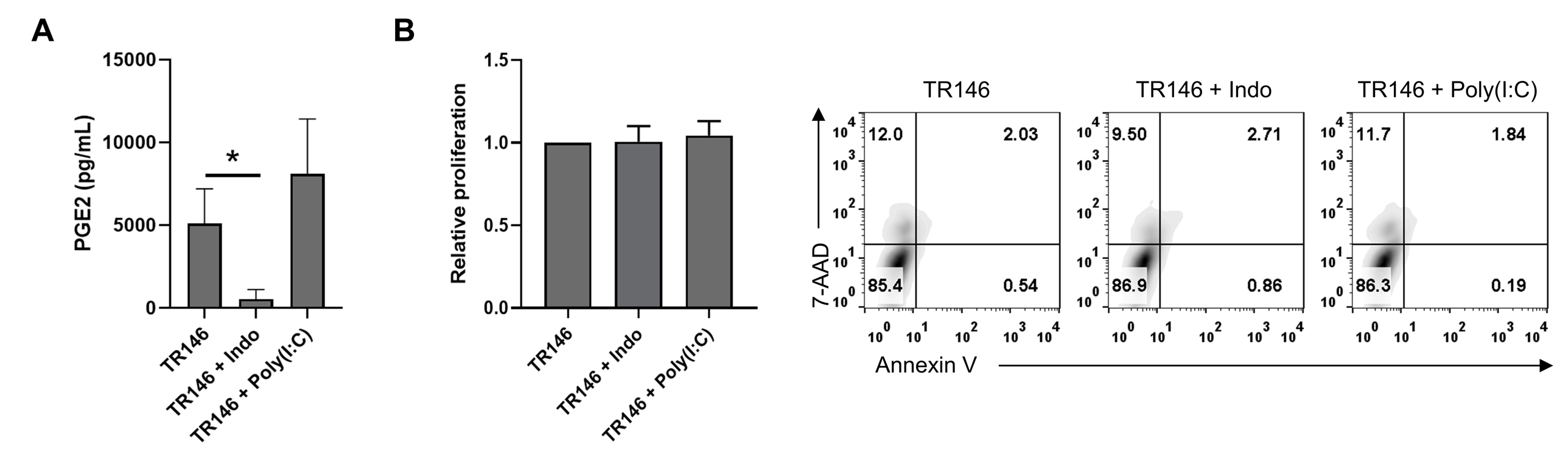

Supplement: Supplementary Figure 2 — Effect of indomethacin and poly(I:C) treatment on TR146 cells. TR146 cells were treated with 2 μg/mL indomethacin (Indo) or 20 μg/mL poly(I:C) for 48 hours. (A) PGE2 release was measured by ELISA in cell-free supernatants. (B) Cell proliferation was measured by MTT assay (left) and viability was analyzed by flow cytometry, staining cells with 7-AAD and Annexin V, as shown in density plots from a representative experiment (right). FACS gates were adjusted by the use of a mouse anti-IgG-FITC antibody (for Annexin V) and unstained cells for 7-AAD. Bar graphs display mean values with SEM error bars. Statistically significant differences (p < 0.05) were noted as “*”. Data were obtained from a total of three independent experiments using samples from different donors. [file Image_2.jpeg]

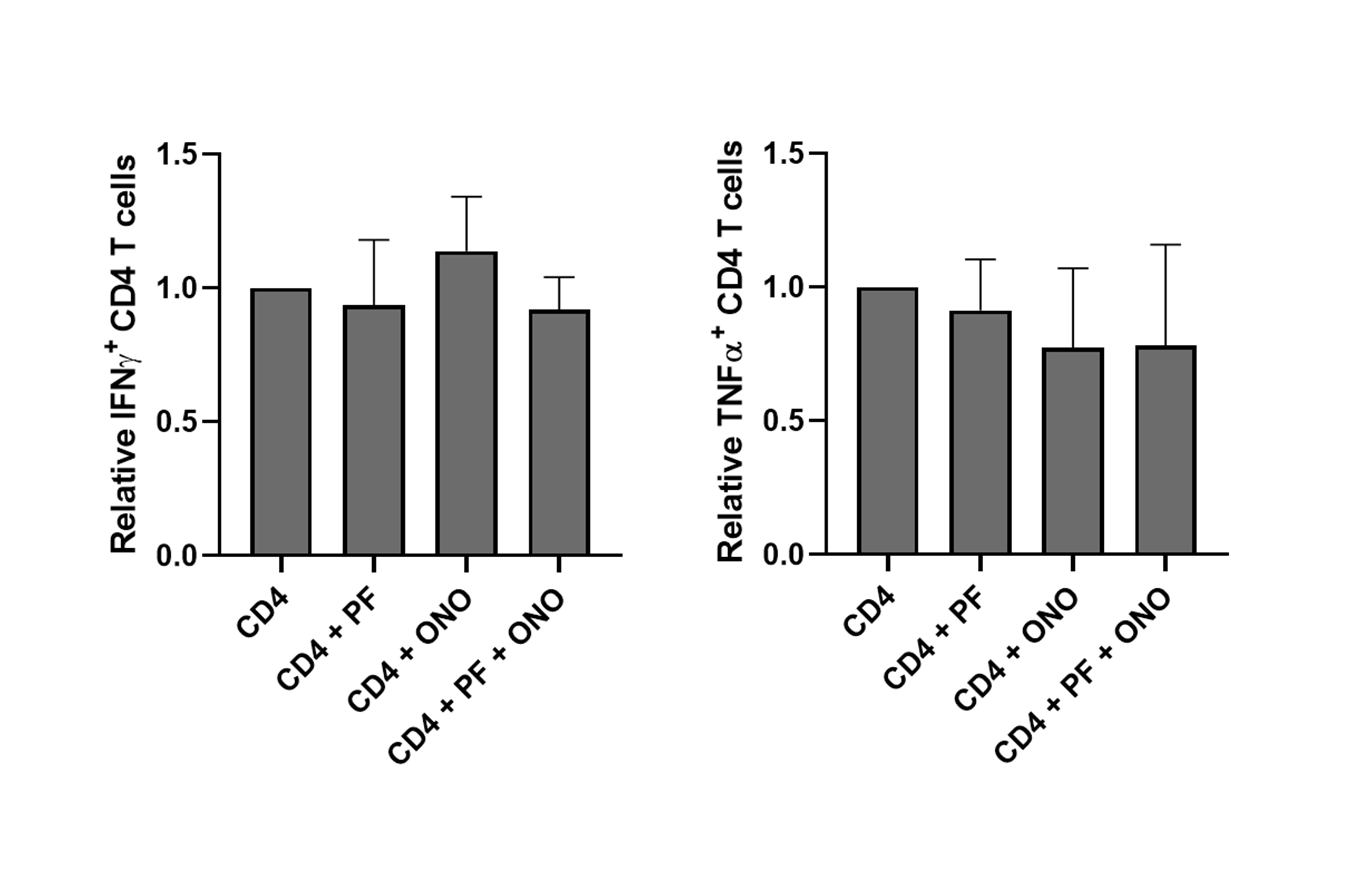

Supplement: Supplementary Figure 5 — Treatment of CD4 T cells with PF-04418948 and ONO-AE3-208 inhibitors do not alter IFNγ and TNFα production. CD4 T cells were treated with PF-04418948 (PF) and/or ONO-AE3-208 (ONO) inhibitors for 1 hour, activated with anti-CD3/CD28 beads and cultured alone for 4 hours. Data were collected by flow cytometry and shown as the amount of IFNγ and TNFα-producing CD4 T cells relative to untreated CD4 T cells. FACS gatings were adjusted by the use of mouse anti-IgG-PE and anti-IgG-APC antibodies. Bar graphs display mean values with SEM error bars. Data were obtained from a total of three independent experiments using samples from different donors. [file Image_5.jpeg]
